# The Dog–Guardian Relationship and Its Meanings: Perceptions, Expectations, and Impacts on Guardians’ Lives

**DOI:** 10.3390/ani16030523

**Published:** 2026-02-06

**Authors:** Tatiane Aparecida de Castro, Carlos Alberto Pegolo da Gama, Denise Alves Guimarães, Igor Tadeu Assis, Marco Aurélio Pereira Horta, Paulo Henrique Araújo Soares, Vinícius Silva Belo

**Affiliations:** 1 Central-West Campus Dona Lindu, Federal University of São João del-Rei, Divinópolis 35501-296, Brazil; tatycastro01@hotmail.com (T.A.d.C.); carlosgama@terra.com.br (C.A.P.d.G.); deniseguimaraes@ufsj.edu.br (D.A.G.); igorassis98@gmail.com (I.T.A.); paulo.h.soares.2007@gmail.com (P.H.A.S.); 2 Laboratory of Hantavirus and Rickettsias, Oswaldo Cruz Institute, Oswaldo Cruz Foundation, Rio de Janeiro 21040-360, Brazil; marco.horta@fiocruz.br

**Keywords:** human–animal relationship, qualitative research, dog, one health

## Abstract

Understanding the relationship between dogs and their guardians provides insight into the role that animals occupy within households and families and, by extension, within society. This perspective offers essential elements for informing policies and practices that affect outcomes in Public Health and animal welfare. This study aimed to comprehensively examine guardians’ perceptions, including expectations, meanings, and social roles attributed to dogs, in a municipality in Brazil. We conducted a qualitative and descriptive investigation grounded in Social Constructionism and Laurence Bardin’s Content Analysis, based on 40 semi-structured interviews. The findings indicate that guardians frequently describe dogs as “family members,” “sources of joy,” “companions,” or even a form of “therapy,” underscoring strong emotional bonds and noteworthy psychosocial benefits. At the same time, the interviews revealed neutral or negative perceptions of this relationship, as well as expressions of grief following the loss of an animal and indignation toward situations of mistreatment. Taken together, the results suggest that although dogs are widely viewed in a positive light and often regarded as family members, divergent understandings of their value and roles persist, reflecting cultural tensions that influence both human and animal well-being.

## Simple Summary

Exploring the relationship between people and their dogs is important because this bond directly influences animal welfare, public health, and family dynamics. This study investigated how dog guardians perceive their relationship with their animals in a Brazilian municipality and how meanings such as care, responsibility, affection, and protection are constructed in everyday life. Using interviews with dog guardians, the study identified that dogs are often viewed as family members, which strengthens emotional bonds but can also lead to misunderstandings about animals’ real needs. While many guardians demonstrate strong commitment to care, gaps in knowledge about responsible guardianship, health risks, and animal behavior were observed. These findings highlight the importance of educational actions led by veterinary and public health professionals to promote responsible care, prevent neglect, and reduce risks to both animals and people. By improving communication between professionals and the community, it is possible to strengthen healthy human–animal relationships and contribute to better quality of life for animals, families, and society as a whole.

## 1. Introduction

Archaeological evidence indicates that the earliest known interaction between humans and dogs dates back approximately 12,000 years. However, this relationship is believed to have begun much earlier, in prehistoric times, when human ancestors started sheltering wolf pups that wandered around their campsites [1]. As humans adapted their environments to meet fundamental needs, dogs emerged as a resource for protection, thermal comfort through body heat, engagement with wildlife, food acquisition, transportation, and other purposes [2,3]. From a sociocultural perspective, these interactions were culturally mediated, as human relationships with non-human beings and the environment were shaped by symbolic meanings, subsistence practices, and social organization [3].

Over time, humans gradually intensified their coexistence with canids, and beyond practical needs, emotional bonds between the species became increasingly refined [3,4]. It is believed that with each new litter, humans selected not only animals that met utilitarian demands but also those with behavioral traits that fostered mutual understanding [1]. This historical process contributed to the incorporation of dogs and cats as central species in human–animal relations [5,6], and they are often regarded today as members of contemporary family structures [7,8]. In many households, dogs are not only companions but are socially positioned as kin, emotional supports, and beings toward whom moral responsibilities and everyday obligations are directed. This transformation of animals from functional resources to affective family members reflects broader social changes in how households construct kinship, care, and emotional attachment to companion animals [8,9].

Currently, roughly half of Western households have companion animals, and positive impacts of the dog–human relationship across different stages of life have been reported [10]. Alongside these benefits, however, other issues have been noted, including zoonotic infections and allergies [2], bite-related risks, and the development of animal-related phobias [2,11]. Studies have also highlighted the economic burden of animal care, constraints on mobility, and psychosocial challenges associated with pet ownership [2,5]. Moreover, behaviors observed in human societies that violate animal dignity remain prevalent. Despite the growing emotional integration of animals into family life, relationships are not always ethical or environmentally responsible, revealing persistent tensions between affection, control, neglect, and exploitation [12]. Within this broader framework, understanding how guardians make sense of these ambivalent dynamics is therefore essential to reveal the everyday practices, values, and expectations that shape human–animal coexistence.

The literature indicates that human–animal interactions influence not only individual physical and mental well-being, but also family dynamics, community connectedness, animal welfare, and public understanding of human–animal relationships, highlighting the multifaceted social impacts of everyday coexistence with companion animals [13]. However, most evidence on these dynamics originates from high-income settings, with studies from developing countries remaining scarce [14], limiting the understanding of how sociocultural and environmental contexts shape human–animal relationships. Thus, examining these interactions in diverse sociocultural settings is important to capture context-specific meanings, practices, and challenges that directly influence coexistence between humans and companion animals, particularly through in-depth qualitative approaches that remain underrepresented in the literature [13]. Taken together, these interlinked human, animal, and environmental dimensions also warrant the use of a One Health framework to investigate companion animal relationships within broader systems of health, care, and coexistence [15].

Given this context, gaining a deeper understanding of the guardian–animal relationship is essential, as such understanding sheds light on the roles animals occupy within families and households and, by extension, within society, particularly in developing-country settings where sociocultural, environmental, and health dynamics may differ substantially from those described in high-income contexts. Accordingly, this study aimed to explore and interpret how dog guardians socially construct the meanings, roles, and expectations of their relationship with animals, as well as how these perceptions intersect with psychosocial well-being, animal welfare, and public health within a One Health context. By focusing on everyday experiences of care, obligation, emotional attachment, and moral reasoning, this study advances understanding of the complex relationship between guardians and their dogs by considering not only emotional bonds but also the social impacts of this coexistence from multiple perspectives. The analysis explores guardians’ perceptions, expectations, meanings, and the significance they attribute to their relationships with their animals. By adopting a qualitative approach, the study offers insights that may support the development of more effective public policies, particularly in areas such as zoonosis prevention, animal welfare, and human health promotion in settings where humans and animals live closely together.

## 2. Materials and Methods

This descriptive qualitative study adopted Social Constructionism as its theoretical framework, based on the premise that knowledge is socially constructed [16]. Qualitative research seeks to understand social reality in its complexity, focusing on the meaning and intentionality of human actions rather than their quantification. This approach enables researchers to grasp how individuals construct and attribute meaning to their experiences [17].

**Figure 1:**
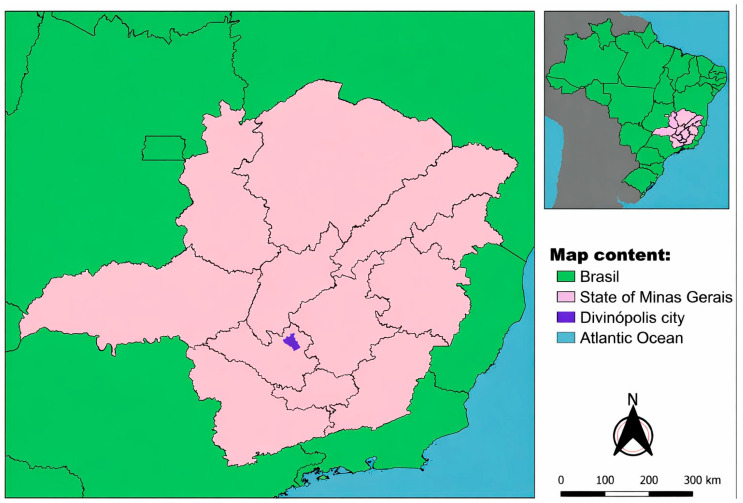
Study area showing the location of Divinópolis municipality in the state of Minas Gerais, southeastern Brazil.

Data were obtained through semi-structured interviews [18] conducted with dog guardians living in the municipality of Divinópolis, Minas Gerais, Brazil (Figure 1), a city located in the central-western region of the state, with a territorial area of 708.115 km^2^ and a demographic density of 326.35 inhabitants per km^2^ according to data from the Brazilian Institute of Geography and Statistics (IBGE). The estimated population is approximately 242,000 inhabitants based on 2024–2025 projections. The municipality presents high school enrollment rates among children aged 6–14 years (98.77%), a municipal Human Development Index of 0.764, an infant mortality rate of 10.65 deaths per 1000 live births in 2023, and a Gross Domestic Product per capita of R$ 40,414.09 (IBGE). The study was conducted across different neighborhoods of the municipality, which generally share similar social and demographic profiles but exhibit substantial internal sociodemographic variability. These characteristics allow for the investigation of diverse environmental, cultural, and socioeconomic factors within the urban context.

Laurence Bardin’s Content Analysis [19], specifically thematic or categorical analysis, was used as the methodological framework for data interpretation. This technique comprises three stages: (1) pre-analysis; (2) material exploration, categorization, or coding; (3) treatment of the results, inference, and interpretation.

All interviews were audio-recorded and transcribed verbatim in full. They were conducted by two researchers trained in public health and qualitative research (TAC and ITA). Transcription began immediately after the first interviews, enabling early familiarization with the corpus and supporting an iterative process of data collection and preliminary analysis. After transcription, each interview was assigned a sequential identification code (E1–E40). The final transcribed corpus comprised 413 pages. Transcription, initial coding, and preliminary analyses were conducted by TAC.

After completing the transcription process, the pre-analysis stage involved organizing the material and systematizing preliminary ideas. This stage included a floating reading of all transcripts and reflections on the objectives and hypotheses. The subsequent material exploration consisted of systematic coding and categorization of meaning units, followed by the construction of the analytical text, treatment of results, inference, and interpretation. To increase traceability, analysis followed an iterative coding process in which codes were progressively refined, as additional interviews were transcribed and examined. Coding combined inductive codes emerging from the data with deductive codes informed by the interview guide. Codes and categories were revisited and revised throughout analysis, and interpretative decisions were documented through analytic notes (memos), creating an audit trail from raw transcripts to consolidated themes. Potential researcher influence on data production and interpretation was addressed through analytic memos and regular team discussions reviewing codes and emerging themes.

Participants were selected from a sample derived from a previous study involving 501 dog guardians, which evaluated, among other aspects, the effectiveness of an educational strategy and animal registration in promoting responsible guardianship and maintaining dogs within households [20].

A type-variety sampling approach was adopted, using the intervention groups defined in the previous study as distinct participant types. In that study, individuals were allocated into four groups according to the intervention received: Group I (questionnaire only—control group); Group II (questionnaire plus educational intervention); Group III (questionnaire plus animal registration); and Group IV (questionnaire plus animal registration plus educational intervention) [20]. This strategy allows the intentional inclusion of participants who differ across relevant variables while sharing a common characteristic, referred to as fundamental homogeneity [21].

No fixed number of participants per type was predetermined. Interviewees were selected through random drawing among those who met the inclusion criteria: participation in the previous study, being a dog guardian, and being over 18 years of age. No refusals occurred among the invited participants. Data collection proceeded until thematic saturation was achieved, defined as the point at which additional interviews no longer contributed new relevant information [22].

The interview guide initially consisted of nine guiding questions organized into three axes: relationship with the animal; animal care; and public health and society. To gain a broader understanding of guardians’ perceptions of the guardian–dog relationship, the interviews also explored additional aspects that shape and intersect with this relationship and its implications for both public health and guardians’ lives, including psychosocial dimensions. Interviews lasted approximately 7 to 40 min.

A total of 40 semi-structured interviews were conducted between April and August 2023. The data were organized into three analytical categories: guardian–animal relationship; responsible guardianship; and public health and society. The present study focuses on the category “guardian–animal relationship” and its corresponding thematic units: guardians’ perceptions of the dog; the animal’s role within the household or family; psychosocial aspects; mistreatment and cruelty toward animals; and experiences of loss. This thematic separation was adopted to facilitate a clearer understanding of the diverse contexts explored and their implications. Themes were consolidated, reviewed, and refined through regular team discussions involving all authors, supporting consolidation of the thematic structure and reflexive consideration of alternative interpretations.

## 3. Results

### 3.1. Characterization of the Sample

Forty semi-structured interviews were conducted, each corresponding to one household, with one dog guardian representing each family. The overall profile of the participants was diverse. Regarding sex, 33 interviewees (82.5%) were women and 7 were men. The minimum age was 19 years and the maximum age was 79 years. Nine (22.5%) households included at least one child. In terms of educational level of the person with the highest level of schooling in the household, complete higher education predominated (55.0%), followed by complete secondary education (27.5%). Table 1 presents the characterization of the interviewees.

In most households, there was only one dog. Overall, 47.5% of households had more than one dog. Among the dogs, 16 (40.0%) were neutered and 24 (60.0%) were not. Regarding how guardians acquired their dogs, most reported having received them as a gift (52.5%), followed by those who purchased a dog (25.0%), picked up a dog from the street (10.0%), had a dog born at home (7.5%), adopted a dog at an adoption fair (2.5%), or adopted from a shelter (2.5%).

### 3.2. Guardians’ Perceptions of Their Dogs

The guardian–dog relationship emerged as multifaceted and complex. Interviewees attributed a wide range of meanings when asked what their dog represented to them. Two broad and contrasting narrative patterns were observed: one emphasizing emotional centrality and family integration, and another stressing clear boundaries between humans and animals.

For many participants, the dog was described as a family member and, in some cases, as a substitute for children or close relatives, reflecting a strong affective bond and high emotional investment. In these accounts, dogs were framed not merely as companions but as integral figures within the household’s emotional structure, as illustrated in the following statements:

*“Well… you know, we think of her as… she’s like a member of the house, of the family, because she’s been with us for almost 13 years now, and she came here as a tiny baby.”*

*(E5)*

*“Ah, Pipoca today is… he’s part of the family, right? Especially for the girls. Without him… well, he must be about 15 years old now, more or less.”*

*(E23)*

*“Mical is the little sweetheart of the house, Mical is our little daughter here, poor thing, our little girl, right Mical? We don’t have children here.”*

*(E14)*

Beyond family integration, some narratives attributed an even greater centrality to the dog, portraying the animal as a primary source of emotional support and meaning in daily life. Expressions such as “everything” or “more important than people” revealed relationships in which the dog occupied a dominant emotional position, particularly among guardians experiencing loneliness or social isolation. As one participant explained, the dog was “everything” in her life.

*“He’s… everything! (laughs). I… gosh, I even get emotional* (the interviewee begins to cry; a moment of silence follows)… *I’m alone, and I stay with her, I talk to her, those things…* (very emotional, crying while speaking about the dog). *Everything.”*

*(E35)*

In contrast, other guardians explicitly rejected the humanization of animals and emphasized symbolic and practical boundaries between people and dogs. While acknowledging affection and companionship, these participants framed the dog primarily as an animal or pet, rather than a family member.

*“Well… I’m not that kind of person who’s like ‘oh, it’s like a child’, that kind of thing. She’s a little animal that really brings harmony to the house, everyone likes her, plays with her, you know? But that’s it… to me she represents an animal, really, but one that brings joy and harmony to the house. That’s it.”*

*(E8)*

*“We’re very clear about this: an animal is an animal, a dog is a dog, and people are people. We separate that very calmly. It doesn’t mean the dog is neglected, but we make that distinction very clearly.”*

*(E12)*

*“There have to be limits, of course. An animal is an animal, a human being is a human being… People calling dogs their kids… I think that’s going too far, right? An animal is an animal. A human is a human… I see a lot of people saying ‘my daughter’… People… I don’t know. That’s how I see it.”*

*(E34)*

### 3.3. The Dog’s Role Within the Household and Family

The role the dog occupied within the family also varied considerably. Beyond emotional presence, interviewees described multiple functions that companion animals may fulfill in society, such as company, fun, protection, and even therapy. Participants described dogs not only as sources of companionship and affection, but also as active contributors to household routines and perceived well-being.

For many guardians, dogs were primarily framed as companions, particularly in contexts of loneliness, daily absence of family members, or reduced social interaction. In these narratives, the dog functioned as a constant presence that mitigated solitude and provided emotional continuity in everyday life. One participant emphasized that the dog was a “companion” who remained with her while her children were grown and away from home:

*“A companion… I think he’s a companion, yes. Because I’m more alone now—my boys, all my boys are married, I only have one son, and I work all day, right? So I stay here at home with them, my dogs and my cats, and I won’t give them away to anyone.”*

*(E6)*

Another recurrent role attributed to dogs was that of protector or guardian of the household. Several interviewees highlighted feelings of increased safety associated with the dog’s presence, particularly through barking and vigilance:

*“He’s very dear, and he’s also our protector, right? We feel much safer with him here… Because anything at all, she barks, you know? We just feel safer, even leaving the girls here alone…”*

*(E20)*

*“Actually, when we got him, it was because I raise birds… and the cats were catching the birds, so we got him to guard them… and he really guards. When he’s here, the cats don’t come close. He became part of the house (laughs).”*

*(E23)*

Alongside companionship and protection, many guardians emphasized the dog’s emotional and therapeutic contributions. Dogs were portrayed as sources of joy, calm, comfort, and emotional regulation, sometimes explicitly compared to a “psychologist” or emotional support figure within the household. For instance, one participant described how the dog brought happiness during the pandemic and encouraged play and positive moods at home:

*“He came at a very good time, during the pandemic, and the girls had someone to play with… I always say he’s not just a protector, he’s a companion… When they see Duque, they get happy. And when you open the door, he comes with the little ball in his mouth for you to play with (laughs).”*

*(E20)*

In contrast to these supportive and affective roles, some accounts foregrounded the practical burden associated with caring for dogs, often expressed humorously but pointing to everyday labor and disruption:

*“Making a mess (laughs), peeing everywhere.”*

*(E4)*

*“To give us work (laughs)… No (laughs). But Nina’s role is to give work, you know? (laughs). But she brings joy too… today she was clean, perfect… then she rolled in the red dirt, you saw, right? (laughs). I always say: Nina’s role is to give work (laughs). But she does bring joy, really…”*

*(E8)*

Overall, these accounts reveal a spectrum of roles attributed to dogs within households, ranging from emotional companionship and protection to sources of daily labor and responsibility, frequently combining both positive and demanding dimensions in the same relationship.

### 3.4. Psychosocial Aspects of the Guardian–Dog Relationship and Its Impact on Daily Life

To further analyze the psychosocial dimensions of this relationship, participants were asked an open-ended final question about whether having a dog brought benefits to their lives, and, if so, which ones. Responses revealed both transformative and burdensome dimensions, with dogs shaping routines, emotional well-being, and household decisions in markedly different ways. Overall, participants’ accounts revealed that living with dogs involved continuous negotiation between emotional connection and the material, temporal, and psychological demands of care, with adjustments to daily life sometimes experienced as beneficial and at other times as burdensome or stressful.

For some guardians, the presence of dogs led to concrete changes in life choices and everyday practices, particularly regarding housing, mobility, and family planning, citing examples such as “choosing where to live,” “being present in meaningful moments,” and “affecting the places they go.”

*“They’re so, so… we were going to move to an apartment. Many apartments came up for us. And we said: we’re not moving because we have two little dogs, and they’re not allowed…”*

*(E1)*

*“It’s everything, right? I’m even planning—doing, how do you say… preparing my birth plan—and I want to start labor at home, with the dogs here… they’re so affectionate… And when the baby arrives, the first thing I want is to show the baby to them… they’ll be together with the baby.”*

*(E4)*

At the same time, many participants emphasized the practical demands associated with caring for dogs, including time investment, daily labor, and financial costs. Guardians frequently referred to cleaning, feeding, and supervision as ongoing responsibilities, as well as to the high expenses related to food and veterinary care.

*“…It’s just that the work is too much, because the mess, my goodness. If it were up to me, I wouldn’t even have one. I like them and all, but if I had to choose not to have one, I wouldn’t, because it’s a lot of work, a lot of mess, it’s work, and it costs too much as well, right.”*

*(E17)*

*“More work for us (laughs)… More work for us… because taking care of a dog isn’t easy, it really isn’t. That’s why a lot of people throw them away, right? Besides being expensive, it’s a big expense, because if you take proper care, you know, like I told you, the best food, the right one for their age… It makes, makes… makes a huge difference in cost, so it’s something expensive to have…”*

*(E19)*

In several accounts, economic strain shaped how guardians evaluated the relationship and their capacity to continue keeping animals. One participant detailed how the cost of dog food competed with basic household needs and personal health expenses:

*“Yeah… for me, since I don’t have the means, I can’t handle going back and forth anymore, so I’m going to end everything, stay without anything… No… I don’t want any animals anymore. My husband travels a lot, we could even get rid of the chickens, just to have a little peace, because it’s too much… I leave early to work, my boys leave early too… the girl, my daughter, goes to school after lunch, I don’t have spare time… You have to buy those big sacks of dog food, so it gets expensive for her, you know? It’s a lot. It’s hard… Because when she was born, she didn’t buy dog food, no, she only bought it for the older ones… and milk for the little one. And now it’s getting tighter, now it’s gotten a bit more difficult… Girl… I need to fix my teeth, and I can’t afford it, so I want to cut back a bit, to see if I can save some money for myself. Because buying a bag of dog food, 120 reais, that really squeezes you… for someone who pays rent, it’s not easy.”*

*(E22)*

For a smaller group, the accumulation of demands transformed the relationship into a source of stress rather than perceived benefit. One guardian described initial enthusiasm followed by frustration as the dog grew, caused damage, and generated ongoing expenses, leading to the decision to not keep animals anymore:

*“At first we thought it was really good when he was tiny, so it was a party, right? But then he grew too much and it got very costly… I don’t want any more… I put a little flower pot here, he would grab it and walk around with it, scattering dirt all over the house, I was about to go crazy. At that moment I got very stressed with him because he was giving so much trouble… if there wasn’t meat, he wouldn’t eat… I kept worrying that he was hungry… I bought the best dog food and he didn’t want it.”*

*(E34)*

In contrast, many participants framed coexistence with dogs as emotionally meaningful, frequently highlighting feelings of peace, joy, comfort, and reduced loneliness. There were accounts from interviewees who described that having a dog had “changed my life” and “transformed my home”:

*“…Look, to tell you the truth, I even became a better person, you know, because they’re a kind of love that nowadays sometimes you expect from humans and you don’t find, you know… Yeeah…* (silence)… (commenting and laughing about the little dog that woke up)… *ah, she has only brought peace into my life, you know… taking care of them properly is hard, like a child, right, but I do all of it and I don’t regret it for a second. I can’t see my life without them. If I go out, I come back quickly because they’re here, you can’t leave them in the dark, you have to come back… you know… it’s like this, they’ve only brought good benefits to my life and to my husband’s life too. He changed a lot; he gets home already thinking of them, so he arrives more relaxed, you know? I can’t see my life without them. I know I’ll suffer a lot if one day I lose them.”*

*(E21)*

Guardians also described sensations and feelings such as “it makes me feel good,” “distraction and joy,” “peace,” “it doesn’t let me feel alone,” and “security”:

*“But that’s what I’m telling you… I think he brings… peace. If you’re sad, the dog cheers you up, you arrive and you’re well received. A dog never greets you like a human being does, because humans sometimes treat you well, sometimes treat you badly… animals don’t have that, you know? A dog doesn’t make that difference. He won’t greet you well only if he’s sick. Then you arrive and he won’t wag his tail, won’t do anything… but if he’s fine, that problem doesn’t exist. Like people say, an animal’s love is always true… a person can be fake, but a dog, or any other animal, isn’t. So you’re welcomed, he doesn’t pretend… his love is one kind only. So that’s it.”*

*(E7)*

*“Oh, she brings joy, right? She brings life to the house. Sometimes when I’m kind of sad, and she comes and plays with me, I get happy, right? So she’s like emotional support, really.”*

*(E28)*

These emotional effects were also reported in relation to other family members, particularly children and older adults:

*“…now that we have a little daughter, for a child it’s good, right, to have a relationship with an animal. That adds to our home too, the importance of having an animal, right? I think it makes things more loving, more affectionate, it builds empathy in the child, especially. I think that’s important for that…”*

*(E12)*

*“Of course it does you good. If I didn’t have them… I’d feel much more alone, I’d be thinking about things… that we shouldn’t be thinking about.”*

*(E6)*

A distinct set of narratives explicitly linked dogs to coping with mental health challenges. Guardians described animals as supporting daily functioning during episodes of depression and anxiety by encouraging routine, physical activity, and emotional engagement. One participant credited the dog with helping her navigate depressive symptoms and regain motivation, while others emphasized how dogs prompted movement and interaction during periods of anxiety or low mood:

*“As I said, he came into a very bad phase for me and my family, not just for me. I started treatment for depression and anxiety… And since then, after I started that treatment, he came into my life and everything changed… it changed little by little… I stopped treatment, thank God I started overcoming depression and anxiety… now everything came back again and I’m doing treatment again… But he helps me so much here inside the house, so much, because there are days when I’m alone here and he’s the one with me; there are days when I wake up and he comes to wake me up… so I wake up happy, I wake up well, in a good mood, wanting to do things, because depression is a very bad thing, a horrible thing to have in your life… he helped me a lot. A lot, really.”*

*(E10)*

*“Yes, that’s exactly it, he helps me overcome things; that’s the right word, he’s been helping me a lot.”*

*(E13)*

*“Oh, it improved a lot, because I was diagnosed with generalized anxiety disorder, and the dog helps because whether you want to or not you have to get up, he asks you to run after him, so you do a bit of exercise. Sometimes you don’t want to get out of bed, you’re kind of sad, and the dog wants, I don’t know, to eat, or the door is closed, so you have to get up, you have to go. He has a pet monkey, he has a pet monkey (laughs), and he brings the monkey to us to play, you feel sorry and you get up and play with him, so it kind of lifts your mood, right? So he helps a lot in the whole health–illness process.”*

*(E16)*

### 3.5. Animal Mistreatment: Perceptions and Legal Aspects

Participants were initially asked whether they were aware of situations involving animal mistreatment. Most reported familiarity with such cases, either through direct observation in their neighborhoods or indirectly through media exposure, particularly news reports and social networks.

Some guardians emphasized that mistreatment was frequently visible in their daily surroundings including abandonment in local streets, while others referred primarily to what they had seen in the media:

*“Not in my own circle… we see it on TV, in the news, right… but I’ve never seen it myself.”*

*(E19)*

*“Wow, it’s what we see the most, right? Mistreatment.”*

*(E25)*

*“Oh, we see a lot of images, right? People abandoning them. Right here on this street, they abandon dogs a lot, wow, we see it all the time.”*

*(E29)*

A subset of participants recounted concrete experiences of abuse witnessed personally, often described with strong emotional reactions. These narratives included physical violence, neglect, and killing of animals by neighbors or acquaintances, reinforcing the perception that mistreatment was not merely a distant phenomenon but part of everyday community life:

*“Before, when I lived here, there was an old man who lived there, he mistreated a little black puppy… After he died, it stopped… And the man who lived downstairs, I don’t know if he still lives there, he drank and beat a little black dog… the dog cried, and I don’t know what he did, he killed the dog. Like I have a neighbor at my house, he can’t drink because he beats his dogs, he has two pit bulls and another regular dog, and he treats the pit bulls and gives only milk and water to the other dog (inaudible).”*

*(E22)*

*“There was one I saw, I don’t know if it was a woman, an older lady who took her somewhere and tied her to a post… or it was a man who tied her up, and wow, he beat the dog, the little dog… oh my God, I felt so sorry, my goodness… (speaks emotionally).”*

*(E35)*

Several guardians perceived an increase in abandonment and mistreatment during the COVID-19 pandemic, linking this trend to social disruption and heightened insecurity. One participant described the appearance of numerous abandoned dogs in the neighborhood during this period:

*“During that COVID epidemic thing, they abandoned a lot of dogs… so many dogs… too many. Look, there at the butcher shop on this street… there are four older dogs there, farm dogs, from people, farmers, who left them here in the neighborhood. They stay there, and the neighbor even fixed up a little shelter for them to sleep, gives them meat, water, food, everyone takes care of them like that…”*

*(E35)*

Several factors may be associated with different forms of animal mistreatment. Regarding abandonment, one interviewee suggested that concerns about illnesses contributed to the decision to abandon animals during the pandemic:

*“Because sometimes people think they could pass something worse to someone who already had the disease, transmit illness… then they talked on the news about leishmaniasis, said this and that, and a lot of older people got sick first, then young people, kids, everyone, all ages, so people got scared. I think that was it.”*

*(E35)*

When reflecting on mistreatment, participants consistently expressed strong emotional responses, particularly sadness, anger, and moral indignation. Mistreatment was framed as irresponsibility and cruelty toward animals for whom guardianship had been assumed:

*“Oh no, anger, right? It’s terrible, I think it’s very sad.”*

*(E16)*

*“My God! It’s cowardice, irresponsibility from someone who took responsibility for that little dog and didn’t take care of it…” (a dog barks loudly and the interviewee says, “sweetheart, stop,” while calming the dog).*

*(E21)*

Views on legal punishment revealed broad support for accountability and, in many cases, for stricter penalties. Most participants advocated for incarceration or more severe sanctions, arguing that fines were insufficient deterrents, as shown below:

*“So now you went there, right, because I think there should be a bigger punishment, right? And they just go there, pay a fine, and that’s it. They won’t do it again, you can be sure. I think they should be arrested, go to jail, like, stay there for a while.”*

*(E13)*

*“I think… I think there has to be that, even though the responsible people are punished, sometimes they’re not punished like they should be. They don’t go to jail, they pay a minimum wage fine, I’ve seen it many times and that’s it. I think it should be even stricter… It’s valid, yes, for humans and for animals.”*

*(E21)*

*“Jail. Jail. Whoever does that to animals…”*

*(E22)*

However, a minority voice questioned the growing legal emphasis on animal protection relative to human welfare. One interviewee, when asked what he thought about punishment for mistreatment, said he believed it was excessive to value animals more than humans, although he acknowledged the reciprocity of the bond with animals.

*“Dogs are getting more value than humans today, right… No, folks, I think an animal is an animal. Today people even go to jail if they hit an animal, right, I think animals should have value, but not that much. Humans first. But like when I worked there… I worked there five years—wow, whenever a dog arrived, it was everything… There was a woman there, my goodness, she’d take the dogs to the vet, all that… and sometimes a homeless person would come and nobody cared… I don’t know, humans are difficult, right? Someone comes and already… If you try to give someone something, clothes to wear, they don’t want it, they come with bad manners. A dog comes and jumps on you, happy… It’s different. You go to pet them and they come all joyful, jumping… Humans are more complicated.”*

*(E34)*

Participants were also asked whether they would report cases of mistreatment. Responses ranged from immediate willingness to report to hesitation rooted in fear and concerns about personal safety, highlighting contrasting ways of navigating mistreatment situations within the community:

*“Yes. For sure. For sure.”*

*(E21)*

*“If I find it somewhere, in a house, with someone beating a dog, I’m going there… I’m going, I’m going… and if things get worse, I’ll call the police…”*

*(E9)*

*“Well, if I had proof, because if I report without proof, it might come back on me later.”*

*(E20)*

*“Well, I… depending on the person, we get scared, right? Because people are killing nowadays… So if we didn’t have to identify ourselves, right? Then it would be easier.”*

*(E31)*

*“*(silence)*… ah, I don’t know. That’s complicated, right, complicated, very dangerous, you know… Very dangerous. If they find out, it’s very dangerous…”*

*(E33)*

### 3.6. Perceptions of Losing an Animal

When asked about experiences of losing a dog, most participants reported having already faced this situation and described it as emotionally distressing. Loss was commonly associated with intense sadness, suffering, and a sense of emotional rupture within the household.

Some guardians who had not yet experienced the death of an animal anticipated profound suffering:

*“When this one here dies, I’m going to suffer a lot. They tell me, ‘Mom, if this dog dies you’re going to suffer so much…’”*

*(E7)*

*“Oh, so sad, for the whole family, it was really sad. Everyone… yeeah… wow, so very sad… I won’t say it’s like losing a family member, but it’s close.”*

*(E33)*

For many participants, grief initially led to rejection of the idea of keeping another dog. However, over time, several adopted again, sometimes immediately or even choosing animals of the same breed. One guardian described intense mourning followed by eventual acceptance of a new dog brought by a family member.

*“My goodness, what me and my mom fought to save him, we managed… We saved him, he got better, then he got sick again and there was no way, he died. That was really bad, wow, I cried for a week, because he was so small, I don’t know what breed it was, but he was tiny… so cute. I said, ‘I never want a dog again, I don’t want one,’ and then my son came home with this dog, he was this size, a little ball, and we kept him, and he’s still here today.”*

*(E13)*

While sadness and suffering were common in accounts of loss, some interviews reflected the heterogeneity already noted in the relationship and the role animals play. One participant spoke calmly about loss:

*“I had eight dogs. Amarelinha died, and on Saturday one of the little ones died, the motorbike ran over him here… All run over. So…”*

*(E22)*

In a distinct set of narratives, death was framed as a natural and easily accepted part of life, extending to both humans and animals. One participant described accepting loss without mourning, emphasizing emotional detachment after death and focusing attention on the remaining animals:

*“Yes… I’ve lost many. I love them so much, but when they go, I accept it… I accept it very easily, without crying or mourning. I don’t know if it’s because another one comes and you focus on the next one, so there are marks, yes, I won’t deny it, but I don’t stay crying over it. And especially this thing about death… when it’s about our people, not animals, but humans, my mother, my father, relatives, people we love, they have already gone too, and I don’t stay lamenting them either. Death is our sister, I even say that, Sister Death, the older sister is death… So when a dog goes, for me he only matters while he’s here. After he’s gone you remember, you play that over in your mind, but it’s not that big attachment anymore, you let go. It’s funny, right.”*

*(E32)*

One participant described the death of a dog as “one less,” while noting that his wife experienced the loss as similar to losing a loved one.

*“For me… for my wife, right? (laughs), you have to ask her. Because for me (laughs), it’s one less. One less, like that. She’s had dogs since she was a kid, she likes dogs. Wow… for her it’s like losing a loved one. And I’m calmer about it, you know?”*

*(E29)*

## 4. Discussion

The results of this study reveal a complex and heterogeneous set of meanings attributed to the guardian–dog relationship, as well as of the impacts of this coexistence within family and social spheres in a Brazilian municipality. The findings are discussed below in light of the scientific literature, focusing on recurrent themes and contrasting interpretations related to what dogs represent to guardians, the roles they occupy within households, psychosocial dimensions of the relationship, perceptions of mistreatment, and experiences of animal loss. Rather than merely confirming positive perceptions of companion animals, the present findings illuminate how emotional attachment, moral responsibility, everyday care, and power relations are simultaneously constructed within human–dog relationships in a developing-country context.

Regarding guardians’ perceptions of dogs, in line with what was identified in the phenomenological study by Giumelli and Santos [2], positive emotional meanings consistently emerged across narratives, with dogs frequently positioned as sources of closeness, affection, and companionship. Within this context, a dominant representation framed dogs as family members, extending beyond symbolic affection to everyday practices of care and emotional investment. This phenomenon is linked to factors such as shrinking family structures and an increasing number of people living alone who seek companionship in animals [23]. With the rapid development of modern civilization and a growing tendency toward isolation among humans, animals often become the only constant presence in the environment, contributing to emotional balance. Consequently, companion animals are increasingly regarded as part of the family core [2]. The results demonstrate that these familial meanings are enacted through everyday caregiving practices that shape household life.

In this sense, anthropomorphization appeared as a recurrent interpretative pattern, as described by Tatibana and Costa-Val [1] and illustrated by several interview excerpts that attribute to dogs human-like characteristics and social roles. Some participants described dogs as a “child.” At the same time, the human–dog relationship remains structurally asymmetrical. As discussed by Tuan [24], processes of domestication and pet keeping involve not only affection and emotional closeness but also human dominance over animals’ bodies, behaviors, and life trajectories. Even when dogs are perceived as family members or “children,” they remain subject to human control in decisions regarding movement, reproduction, discipline, and daily routines. For Lewgoy et al. [25], the personalization of animals can be observed in their “puppyfication,” most evident in intensified mothering, heightened sensitivity to animals’ needs and care, reflected in the expanding range of veterinary specialties, the “humanization” of their care, and even the creation of institutions to deal with animals’ deaths. By jointly examining affection and asymmetry, the present study highlights the coexistence of intimacy and control as a central, yet often underexplored, dynamic of contemporary human–dog relationships.

People may decide to have a companion animal for several reasons, including the fact that these animals are energetic, interact spontaneously with humans, and show unconditional affection toward their guardians [2]. In this context, a dominant emotional narrative across interviews framed the dog as “everything” and even “more important than people”. For others, the dog’s relevance in their lives was so intense that it was difficult to put into words; still, others associated dogs with positive feelings of joy and happiness. By contrast, other accounts emphasized potential downsides. These contrasting meanings reveal that the human–dog bond is not a uniform emotional phenomenon, but a socially negotiated relationship shaped by affection, ambivalence, and moral evaluation.

It is also important to consider that alongside emotionally intensive bonds, a pragmatic framing emerged in several narratives, in which the animal was described as “just an animal” or simply “a pet.” Another recurring account positioned dog ownership as primarily motivated by the preferences of other family members. To understand an individual’s behavior, Lewin [26] argues that the individual must be considered within the field in which they are embedded and with which they interact. This means that resulting behavior depends not only on internal reality, but also on external reality, and these realities influence one another. Given the diversity of participants’ responses, Giumelli and Santos [2] suggest that companion animals were part of a life field for a subset of guardians that began in childhood; thus, continuing to keep pets throughout life is common for such individuals. The present findings contribute by illustrating how diverse social trajectories and household dynamics shape distinct bonding profiles within the same community. Understanding how guardians assign meaning to the relationship makes it possible to plan actions that are better tailored to different bonding profiles, thereby supporting strategies for responsible guardianship education, zoonosis prevention, and other One Health initiatives that adopt an integrated approach linking human, animal, and environmental health in which the quality of the guardian–dog relationship can be treated as a central element for the joint promotion of human and animal health [15,27].

The place dogs occupy within the family was likewise highly heterogeneous. Beyond the animal’s role in the household, the interviews revealed multiple functions of companion animals in society. Such functions change as societal needs evolve [28]. Accordingly, participants’ accounts indicate that dogs currently fulfill diverse roles, such as “companionship,” “fun,” “protection,” and “therapy.” In a study by Cardoso et al. [23], analysis of reasons for keeping animals showed that 66.7% of dogs were kept for companionship and 26.3% for security, highlighting greater contact between animals and humans. The closeness and high valuation of dogs’ roles in guardians’ lives, made explicit in the interviews, also reinforces a feature of human social evolution: life in less communal and more individualistic societies, which distances individuals from their natural environment of interaction with other species [2]. In this sense, relationships with companion animals may represent a way of seeking contact with the natural world, which was also evident in the present study. It is worth noting that the development of human–companion animal relationships has occurred alongside broader behavioral shifts in society, including fewer children and greater overall resources, assigning companion animals the status of family members and keeping them more indoors than outdoors, and incorporating animal-related expenses into the household budget [12].

Further interviewees’ accounts show that dogs may have different impacts on guardians’ lives, ranging from small day-to-day changes—such as influencing decisions about where guardians go—to major transformations, such as participating in meaningful life events and shaping choices about the type of home in which to live. These narratives indicate that the intensity of emotional attachment directly shapes daily routines, priorities, and life decisions, suggesting that animal health and responsible guardianship initiatives [20] should consider not only practical caregiving behaviors but also the affective meanings that organize everyday coexistence, given that emotional bonds can strongly guide decision-making processes.

Participants also described the clear benefits of having a dog. In the interviews, guardians highlighted dogs’ potential to transform and reshape their lives. Hodgson and Darling [29] emphasize that living with animals promotes a range of benefits for humans, positively affecting life in multiple domains. In a study by Fuchs [30], which sought to understand the psychological meanings attributed to companion animals in everyday life based on reports by owners and non-owners, the author concluded, among other points, that the presence of a pet may bring psychosocial benefits to guardians. Similarly, a qualitative study of dog owners in a rural district of Sri Lanka [14] found that participants perceived stress reduction, mental satisfaction, and enhanced personal and family well-being through interaction with their pet dogs. According to Nieto-Palma and García-Gómez [31], companion animals provide a source of love, affection, and companionship, as well as benefits such as increased feelings of happiness, security, and self-esteem [32]. As indicated in the present study, these benefits are closely linked to the ways in which dogs are incorporated into family life as emotionally significant members, with daily interactions, caregiving routines, and shared experiences fostering companionship, emotional support, and a sense of belonging. In this way, the positive outcomes reported by guardians are not merely individual psychological effects, but emerge from relational practices that resonate with broader discussions in companion-animal scholarship on pets’ integration into family life and everyday caregiving.

Emotional support emerged as a central and recurrent theme across narratives, with dogs functioning as a form of “therapy” in coping with mental health conditions such as depression and anxiety. Companion animals have marked importance due to the benefits their interaction with humans can bring. These benefits include reduced depression, stress, and anxiety; improved mood; greater motivation for healthy activities; increased socialization among older adults and people with physical or mental disabilities; and improved learning and socialization in children [14,33]. This dimension of emotional support can also be interpreted through the lens of affective labor. Drawing on Plourde’s analysis of cats as “healing” and affective laborers in Japanese cat cafés [34], in which animals facilitate relaxation, emotional engagement, and a sense of comfort through sensory and everyday interaction, our findings suggest that dogs similarly contribute to guardians’ affective well-being in domestic life. Beyond passive companionship, dogs in this study actively participated in the production of emotional relief, routine stability, and feelings of security, helping guardians cope with stress, loneliness, and mental health challenges. In this way, affective support emerges as an ongoing relational process embedded in daily human–dog coexistence, rather than merely an incidental benefit of pet ownership.

On the other hand, a contrasting and recurrent theme involved the burdens of care, described as demanding work that requires time, effort, and financial resources. From a broader relational perspective, these responsibilities reflect how companion animals are incorporated into human lives as dependent beings shaped by human authority. While guardianship is often expressed through care and affection, it simultaneously entails regulation, discipline, and control, reinforcing asymmetries of power that structure everyday human–animal coexistence [24]. Another recurring narrative described stress arising both from the responsibilities of animal care and from keeping dogs primarily to meet the emotional needs of other family members. These findings reveal how emotional attachment is inseparable from sustained labor, moral obligation, and everyday negotiation within households. This aspect is relevant because such perceptions may facilitate or hinder adherence to desirable practices, including responsible guardianship and abandonment prevention. Parslow and Jorm [35], using a random sample of 2530 adults aged 40–44 years, found that physical and mental health measures, including use of general practice services, were not significantly affected by owning and caring for companion animals. However, people who owned or cared for pets used analgesic medications more frequently. The authors concluded that owning or caring for pets may not confer health benefits in this age group.

Finally, Almeida et al. [10], in a study on human–animal relationships, highlight that beyond benefits for humans, good coexistence also brings benefits for animals, including better food, housing, leisure opportunities, and sanitary conditions. Dukes [36] note that harmful interactions for both humans and animals may also occur, especially when owners are unprepared to handle companion animals. Such situations may lead to stereotyped behaviors, behavioral despair, and aggressiveness on the animal’s side [36]. For humans, this may translate into dissatisfaction with the animal’s behavior, potential aggression by the animal, and transmission of zoonoses, especially rabies [10,37]. Considering this context and the present findings, the guardian–dog relationship appears to involve a combination of important emotional benefits and significant practical challenges, producing a complex and demanding scenario for public policy development. Advancing strategies that integrate responsible guardianship education, support for guardians, and accountability mechanisms for those who fail to meet basic care duties is therefore essential to reduce harm, prevent abandonment, and ensure adequate conditions of animal well-being [38].

In this sense, it is important to emphasize that dogs are often subjected not only to mistreatment, but also to abandonment or inadequate living conditions. Animal mistreatment practices have been common throughout history and remain so today [39]. Animal mistreatment is understood as neglect in one or more of the following domains: nutrition, environment, health, and behavior. Compromises in these four physical domains are cautiously used to infer affective experiences related to the fifth domain, namely the mental domain [40]. Mistreatment constitutes intentional harm, resulting in animals that suffer physically and/or emotionally—socially unacceptable behavior intended to cause pain, suffering, stress, or death [41]. Ascione [42] and Arkow et al. [43] divide mistreatment into cruelty (active), neglect (passive), or a combination of both. In Brazil, the most common category is neglect, or passive mistreatment, accounting for roughly 80.0% of cases [44]. Notably, definitions of mistreatment were not presented to interviewees, since the objective was to understand their perceptions of the issue. In addition, participants were not asked about the methods they used to train or discipline animals, nor about their views on such practices—for example, whether physical aggression used to educate a dog would be recognized as mistreatment. This aspect should be investigated further in future qualitative studies.

Lewgoy et al. [25] argue that denunciations of cruelty in the treatment of abandoned dogs and cats express a plea for compassion and rights. During the interviews, awareness of mistreatment cases emerged as widespread across narratives, either through personal experience or through media reports. Frequently and effectively disseminated on social media, such denunciations expose vulnerability through images of animal bodies harmed by human action. This exposure signals a moral demand tied to claims for rights of vulnerable beings and to calls for penalization and criminalization of those responsible for animal suffering [25].

An interesting point that emerged during the interviews was participants’ perception of increased awareness of animal mistreatment during the COVID-19 pandemic, for example, through abandonment. A study by Pereira [45], which aimed to analyze the relationship between animal mistreatment and domestic violence in 2020 (the pandemic period), showed that confinement during the pandemic significantly increased aggression suffered by both women and animals.

In the 1990s, the so-called Link Theory became widely known after numerous studies in the United States demonstrated a relationship between animal mistreatment and various forms of violence against people [46,47,48]. This theory is characterized by work identifying individuals’ capacity to act violently, directly or indirectly, toward animals and people, particularly the most vulnerable. Domestic violence, child abuse, and animal cruelty are thus closely connected, and this cycle will persist until somehow interrupted [49].

The data obtained in this study are consistent with findings from Rodrigues et al. [7] on mistreatment and abandonment of animals, in which 94.0% of guardians reported awareness that these acts constitute crimes. An interesting result in Rodrigues et al. [7], however, is that this awareness diverged from responses about animal welfare: only 30.0% of guardians mentioned not mistreating, not harming, not abandoning, and not frightening animals as elements related to responsible guardianship. The authors interpreted this as evidence of population-level misinformation about what responsible guardianship entails, preventing citizens from clearly distinguishing mistreatment from culturally accepted practices in dealing with animals.

Within this context, interviewees were also asked about their views on punitive actions for mistreatment. Despite different feelings about whether to report cases, Santana and Oliveira [12] note that society has shown growing interest in issues related to animal cruelty. Effective monitoring and punishment of mistreatment require that society understand what behaviors constitute this offense and know which institutions are responsible for receiving complaints [50,51]. In this way, the findings point to the need for continuous educational actions capable of clarifying what characterizes mistreatment and guiding guardians about their legal duties, supporting both prevention of new occurrences and the strengthening of qualified reporting [52,53].

Beyond the positive aspects that relationships with animals may provide to humans, negative aspects must also be considered. Among these, losing an animal due to illness, disappearance, or theft can cause intense suffering and distress for guardians, including depression [2]. These feelings were reported by participants. Experiences of animal loss emerged as emotionally salient across narratives, and participants expressed multiple negative emotions. Others who had not experienced such loss described the suffering involved even in imagining it. Experiencing an animal’s death may provoke painful and intense feelings, thus constituting a negative aspect of having a companion animal [2,54]. Barbosa [55] note that the absence of a companion animal may cause sadness, but the way each person experiences this feeling varies. A recurrent coping pattern involved obtaining another animal soon after loss, while other narratives framed death as part of a natural life cycle. Notably, the interviewees who spoke more calmly and naturally about loss were guardians of a large number of dogs and had experienced many losses over their lifetimes. One of the clearest expressions of anthropomorphization emerges in the way contemporary society deals with the death of companion animals. Observing how guardians in this study described grief, loss, and post-death practices reveals that pets may be granted a human-like status within these relationships, although such meanings are often accompanied by emotional tensions and social ambiguities [56].

Several limitations of this study should be acknowledged when interpreting the findings. First, this qualitative investigation was conducted in a single medium-sized municipality, which limits the transferability of the results to other sociocultural contexts, particularly regions with different socioeconomic conditions, urbanization patterns, and practices related to animal care. In addition, participants were selected from a sample derived from a previous study focused on responsible guardianship, which may have favored the inclusion of more engaged or health-conscious guardians. The data relied exclusively on self-reported narratives, which are subject to social desirability bias, especially regarding morally sensitive issues such as animal mistreatment, caregiving practices, and emotional attachment. Furthermore, the lack of complementary data sources, such as direct observation or quantitative information, limits the ability to contrast reported perceptions with everyday practices. Together, these factors should be considered when assessing the scope and implications of the findings. Despite these limitations, the present study contributes empirical qualitative evidence on the social construction of the human–dog relationship in a Brazilian urban context, an understudied perspective in the companion animal literature.

## 5. Conclusions

This study highlights the significant impact that dogs have on the daily lives of their guardians and the affective place they occupy within families and households, with a predominance of perceptions pointing to positive effects of living with companion animals. At the same time, the findings reveal considerable heterogeneity in how this relationship is constructed and in how guardians understand the dog’s role in their lives. Based on a broad reading of the interviews, participants can be grouped into three major categories according to their perceptions: (1) those who view the dog as “everything,” “more important than people,” a source of “therapy,” or someone who “changed my life”; (2) those who see the dog as a “family member,” a source of “joy,” and primarily a “companion”; (3) those who perceive the dog as “just an animal” or “a pet,” whose role is limited to “guarding the house” or “keeping watch.” The intensity of these perceptions also shaped guardians’ emotional expressions during the interviews, reinforcing the emotional depth of the human–companion animal bond and its relevance to social life and public health.

The social and cultural constructions evident in these findings should inform the development of policies and educational programs, emphasizing the importance of culturally sensitive approaches. Understanding guardians’ perceptions, and how Responsible Pet Ownership practices are socially constructed and influenced by the social, cultural, and historical contexts in which guardians are embedded, is essential for the success of such initiatives. By situating these dynamics within a Brazilian sociocultural context, the study provides novel insights into how structural conditions and everyday caregiving practices shape the human–animal bond, with direct implications for integrated One Health strategies. Overall, the study’s findings suggest that public health and animal welfare initiatives must integrate technical and scientific knowledge with an appreciation of the affective and sociocultural realities that shape guardian–dog relationships, including supportive and educational actions aligned with the different bonding profiles, as well as effective mechanisms to reduce neglect and mistreatment.

## Figures and Tables

**Table 1:** Sociodemographic profile of participants and number of dogs per household.

Interview Code	Age (Years)	Sex	Highest Educational Level in the Household	Number of Dogs in the Household
E1	75	Female	Incomplete primary education	2–3
E2	29	Male	Complete secondary education	1
E3	49	Female	Complete higher education	1
E4	35	Female	Complete higher education	4–6
E5	59	Female	Complete primary education	1
E6	64	Female	Complete secondary education	2–3
E7	45	Female	Complete secondary education	4–6
E8	55	Female	Complete higher education	1
E9	66	Female	Complete secondary education	≥7
E10	20	Female	Complete higher education	1
E11	55	Female	Complete higher education	1
E12	52	Male	Complete higher education	2–3
E13	41	Female	Complete secondary education	2–3
E14	53	Female	Complete higher education	1
E15	19	Female	Complete secondary education	1
E16	23	Female	Complete higher education	1
E17	58	Female	Complete higher education	2–3
E18	67	Female	Complete higher education	2–3
E19	45	Female	Complete secondary education	1
E20	49	Female	Complete higher education	2–3
E21	42	Female	Complete secondary education	2–3
E22	45	Female	Incomplete primary education	2–3
E23	42	Female	Complete secondary education	2–3
E24	52	Male	Complete primary education	4–6
E25	52	Female	Complete primary education	2–3
E26	46	Female	Complete higher education	1
E27	58	Female	Complete higher education	1
E28	24	Female	Complete secondary education	1
E29	54	Male	Complete higher education	4–6
E30	53	Female	Complete higher education	1
E31	68	Female	Complete primary education	2–3
E32	64	Female	Incomplete primary education	≥7
E33	79	Male	Complete higher education	1
E34	57	Female	Complete higher education	1
E35	57	Female	Complete secondary education	1
E36	63	Male	Complete higher education	1
E37	46	Female	Complete higher education	1
E38	67	Male	Complete higher education	2–3
E39	76	Female	Complete higher education	1
E40	36	Female	Complete higher education	1

## Data Availability

The qualitative interview data supporting the findings of this study are available from the corresponding author upon reasonable request.
